# Vitamin D status and disability among patients with multiple sclerosis: a systematic review and meta-analysis

**DOI:** 10.3934/Neuroscience.2021013

**Published:** 2021-02-05

**Authors:** Mahmood Moosazadeh, Fatemeh Nabinezhad-Male, Mahdi Afshari, Mohammad Mehdi Nasehi, Mohammad Shabani, Motahareh Kheradmand, Iraj Aghaei

**Affiliations:** 1 Gastrointestinal Cancer Research Center, Non-communicable Diseases Institute, Mazandaran University of Medical Sciences, Sari, Iran; 2 Mazandaran University of Medical Sciences, Sari, Iran; 3 Department of Community Medicine, Zabol University of Medical Sciences, Zabol, Iran; 4 Pediatric Neurology Department, Mofid Hospital, Shahid Beheshti University of Medical Sciences, Tehran, Iran; 5 Neuroscience Research Center, Institute of Neuropharmacology, Kerman University of Medical Sciences, Kerman, Iran; 6 Health Science Research Center, Addiction Institute, Mazandaran University of Medical Sciences, Sari, Iran; 7 Neuroscience Research Center, Guilan University of Medical Sciences, Rasht, Iran

**Keywords:** multiple sclerosis, MS, disability, vitamin D, meta-analysis

## Abstract

Association between the serum vitamin D level and disability of patients with multiple sclerosis (MS) has been investigated during several researches. However, these studies reported different results. The current study aims to estimate the correlation between the concentrations of 25 (OH) vitamin D and the level of disability among MS patients. Using Mesh and non-Mesh terms related to MS, disability level and vitamin D, different data banks were searched. Required information was extracted from the selected eligible primary articles. Stata version 11 software was applied for combining the primary correlation coefficients using random effect model. The effect of MS type and patients' age was assessed using meta-regression models. Sensitivity analysis was performed to investigate the role of each primary study in the pooled estimate. Egger test was applied to find any publication bias. Of 14 eligible studies, the total correlation coefficient (95% confidence interval) between 25 (OH) vitamin D level and disability in both sexes as well as among female was estimated as of −0.29 (−0.40, −0.17) and −0.35 (−0.46, −0.24) respectively. Two articles carried out among male did not report significant results. Our meta-analysis showed a significant negative correlation between 25 (OH) vitamin D level and disability of MS patients so that the disability reduces with increasing the 25 (OH) vitamin D level.

## 1. Introduction

Multiple sclerosis (MS) is a multifactorial, autoimmune and inflammatory disorder in the CNS characterized by demyelination, inflammation, and neurodegeneration which is one of the main causes of disability among young adults [1]–[3]. Clinical manifestations of MS include paresthesia, diplopia, visual impairment, fatigue, emotional instability and hypoesthesia [4],[5]. In developed cases, sever paresthesia affects upper and lower limbs [5]. This disorder has different prevalence in different regions of the word and countries categorized in three zone (low, Medium and high) by Kurtzke. According this classification, countries such as England, Germany, and Switzerland which have prevalence rates between 100 and 150 per 100,000 would likely be ranked as “high” and in the same category as countries such as Canada, Norway, Sweden, Scotland, and Ireland which haven prevalence rates between 150 and 350 per 100,000. Therefore, Wade proposed a five-zone classification system that capable to avoid grouping together countries with prevalence rates which are incomparable [6]. The prevalence and incidence of MS is higher for female than male [7]. Age, sex, familial history, infection, race and environmental factors are determinant factors of this disorder [8]. According epidemiological study, MS is wide spread in the populations in North Europe because people of this area suffered to lack of vitamin D due to decrease of sunlight exposure [1].

Exposure to sunlight and vitamin D level are some environmental factors of developing MS [9]. Vitamin D3 is synthesized from 7-dehydrocholesterol using the ultraviolet in the skin [10]. Vitamin D has an immunomodulatory roll for immune system and different immune disorders such as MS [11]. Vitamin D deficiency and high prevalence of MS has been reported in regions with limited sunlight [12]. Disability in MS patients is measured by Expanded Disability Status Scale (EDSS). This scale is associated with physical function of patients [13].

Several studies have been carried out in the recent years investigating the role of vitamin D level in developing MS. Fahmi et al. reported a correlation coefficient as of −0.755 between 25 (OH) vitamin D level and disability among MS patients which was statistically significant [14]. The corresponding correlation coefficients in the studies carried out by Kutlu [15] in Turkey (r = −0.102) as well as by Nikanfar [16] in Iran (r = −0.08) were not statistically significant. Conversely, Brola et al. in Poland [17] found a moderate and significant positive correlation (r = 0.38).

The above controversies in the results of different studies can be settled by systematic review and meta-analysis. In such studies, the results of primary researches are pooled together and the characteristics of the different studies (location, time, methods) is taken into account [18]. Therefore, these pooled estimates are more reliable with higher accuracy. The aim of the current study is to estimate the correlation between 25 (OH) vitamin D level and the degree of disability in MS patients.

## 2. Materials and methods

Before implementation of the study, the protocol was registered in PROSPERO https://www.crd.york.ac.uk/PROSPERO, ID = CRD42019120422. The systematic review was conducted according to the PRISMA guideline [19].

### 2.1. Inclusion/exclusion criteria

Inclusion/exclusion criteria have been designed based on PICO protocol. P (population) indicates MS patients. I (intervention) and C (comparison) did not apply in this study because of the correlation design of the study. O (outcome) refers to the disability. The primary studies included in this systematic review were cross sectional and cohort. Any study reporting a correlation or association between disability and 25 (OH) vitamin D level among case group (MS patients) would be considered as eligible study. The search was carried out during 26/11/2018 and 3/12/2018. The eligible studies were published from any time to the end of November 2018 in Persian or English in any country.

### 2.2. Search strategy

We investigated all available databanks such as PubMed, Scopus, Science direct, Cochrane, Google Scholar search engine and also Iranian databanks (SID and Magiran) with relevant keywords (Mesh and non-Mesh) using “OR”, “AND” and “NOT” operators in English and Persian languages as below: “Multiple sclerosis” [Title] OR “MS” [Title] OR “Relapsing remitting Multiple sclerosis” [Title] OR “Relapsing-Remitting MS” [Title] OR “ RRMS” [Title] OR “Secondary Progressive Multiple sclerosis” [Title] OR “Secondary-Progressive MS” [Title] OR “SPMS” [Title] OR “Primary Progressive Multiple sclerosis” [Title] OR “Primary-Progressive MS” [Title] OR “PPMS” [Title] OR “Progressive-Relapsing Multiple sclerosis” [Title] OR “Progressive-Relapsing MS” [Title] AND “vitamin D” [Title] OR “25-hydroxyvitamin D” [Title] OR “25 (OH) vitamin D” [Title] OR “25 (OH) vitamin D3” [Title] OR “25 (OH) D” [Title] OR “1,25-dihydroxyvitamin D” [Title] OR “1,25 (OH) 2D” [Title] AND “disability” [Title] OR “Expanded Disability Status Scale” [Title] OR “EDSS” [Title].

In order to increase the number of the primary eligible studies, all identified articles were investigated regarding their references. In addition, some researchers were addressed by email to help us finding any required information. Endnote software was used for management of the references.

### 2.3. Selection of the studies

First, duplicate evidences were excluded. The rest articles were reviewed in terms of titles, abstracts and full texts and irrelevant papers were omitted. Three researchers performed the above steps; two of them in screening the evidences and the third researcher in management of the inconsistencies.

### 2.4. Data extraction

Two expert investigators independently extracted the required information from the selected articles. The extracted data contained identity variables (first author surname, title, journal name, and year of publication, location of the research and language of the article), statistical information (sample size, correlation coefficient between 25 (OH) vitamin D and disability in MS patients) and also variables needed for assessment of “Risk of Bias”.

### 2.5. Risk of bias

Risk of bias was assessed using NOS (The New-Castle-Ottawa Scale) containing three sections (selection, comparability and exposure) with 0–9 scores (four scores for selection, two scores for comparability and three scores for exposure section). All papers with scores less than five were excluded from the meta-analysis. The assessment was conducted independently by two methodologists [20].

### 2.6. Statistical methods

Stata software ver. 11 was used for all statistical procedures. Using equation SEr = sqrt [(1 − r^2)/(n − 2)], standard error of correlation coefficients was calculated for all primary studies. The heterogeneity between the primary studies was assessed using Cochrane test and I-square index. The pooled correlation coefficient was estimated based on random effect model and inverse variance method. Point and pooled estimates with 95% confidence intervals were illustrated in forest plots. Some of the studies had not reported the correlation coefficient. Therefore, P value meta-analysis was applied for combining the significance level of the primary results. The effect of type of MS and age of patients on the heterogeneity was assessed using meta-regression analysis. The correlation between 25 (OH) vitamin D level and disability was also investigated based on the MS subtypes. The role of each primary study on the total estimates was assessed by sensitivity analysis. Publication bias was evaluated using Egger test.

## 3. Results

**Figure 1.**
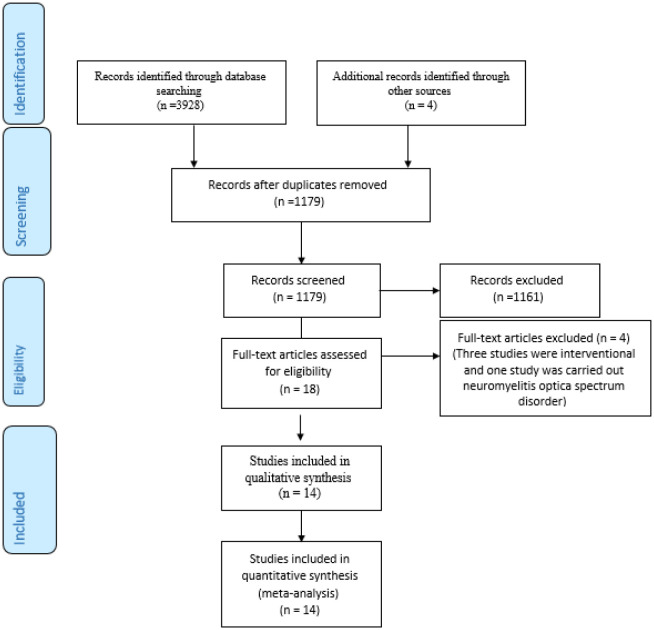
Searching and selection of the primary studies.

The primary search of the mentioned databases revealed 3932 articles declined to 1179 after exclusion of duplicated papers. Assessing the inclusion/exclusion criteria, 1161 studies were identified irrelevant. The full texts of the 18 remained papers were investigated and four studies were omitted (three studies were interventional and one study had been carried out among patients with neuromyelitis optica spectrum disorder) (Figure 1). Finally, 14 studies [14]–[17],[21]–[30] were entered into the systematic review and meta-analysis process (Table 1). According to the Newcastle, all of which had five and more quality scores.

All of the selected studies had been published between 2007 and 2018 in Australia, Brazil, Egypt, France, Iran, Mexico, Poland, Portugal, The Netherlands and Turkey.

Serum levels of 25 (OH) vitamin D were measured in all studies, except for the kaget et al. study, which also measured levels of 1, 25 (OH) vitamin D, but we only analyzed the correlation of 25 (OH) vitamin D and EDSS. In general, less than 1% amount of 25 (OH) vitamin D circulate free hormone and 99% of them bonded with vitamin D-binding protein (DBP) and albumin (85–90% and 10–15%, respectively), but unbound hormone is biologically active [31]. Therefore, we extracted that which method was used for measurements of 25 (OH) vitamin D in each study (Table 1).

19 evidences were extracted from of 14 eligible studies, so that 13 study were carried out among 2477 patients of both sex, four studies conducted among 287 female and two studies performed among 53 males (Table 1). The samples sizes of the primary studies differed from 20 patients in the Rito [26] study to 700 patients in the Karampoor study [24].

13 out of 14 studies reported the correlation coefficient between 25 (OH) vitamin D level and disability, so that 10 studies reported significant negative correlations but only Brola et al. study's reported positive correlations [17]. However, investigating the full text of this study revealed probable mistake. Because they mentioned a negative association between 25 (OH) vitamin D and disability. Therefore, we considered this correlation coefficient as a negative indicator.

**Figure 2.**
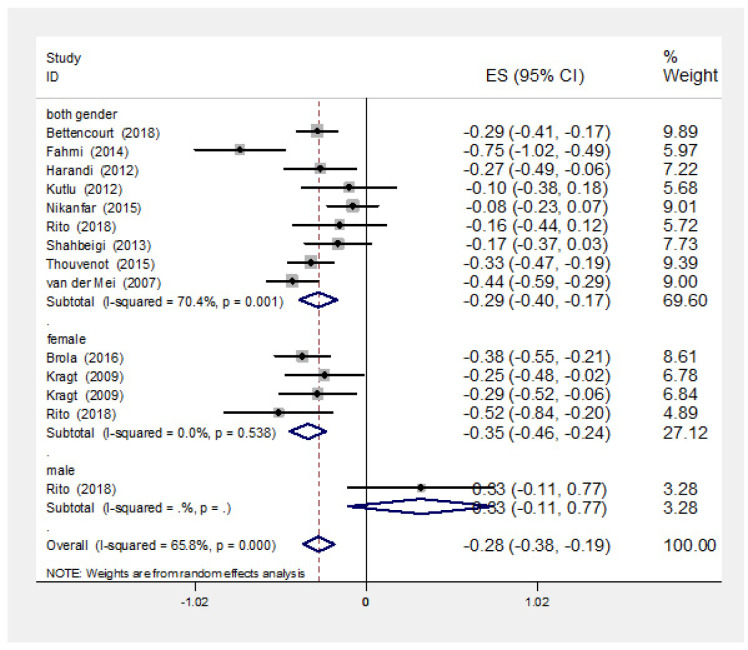
Point and pooled correlation coefficients (95% confidence intervals) between vitamin D level and disability of MS patients based on sex. ES: Effect Size.

9 out of 13 studies, carried out among both sexes, were reported negative correlation coefficients between 25 (OH) vitamin D level and EDSS, but only the five of them were had statistically significant correlation (Figure 2). Furthermore, a significant heterogeneity was observed between the results of these studies (I-squared: 70.4%, Q = 27.02, P = 0.001). Pooled correlation coefficient was estimated as of −0.29 (95% CI: −0.40, −0.17). Because four studies did not report correlation coefficients, P value meta-analysis was conducted for 12 studies to estimate the pooled association (Kragt study was excluded from p value meta-analysis because had not reported exact p value). The pooled P value was less than 0.001.

All of this four studies reported correlation coefficients for female showed negative and significant correlations. No significant heterogeneity was observed between the primary results (I-squared: 0%, Q = 2.17, P = 0.538). The pooled correlation coefficient between vitamin D level and EDSS index for female was estimated as of −0.35 (95% CI: −0.46, −0.24).

Two studies have investigated the association between EDSS and vitamin D level among male, one study had reported positive correlation coefficient which was non-significant. Another study had only reported P value which was not statistically significant.

According to meta-regression models, the MS type (β = 0.11, P = 0.027) and mean patients' age (β = −0.02, P = 0.020) had statistically significant roles in the heterogeneity.

**Figure 3.**
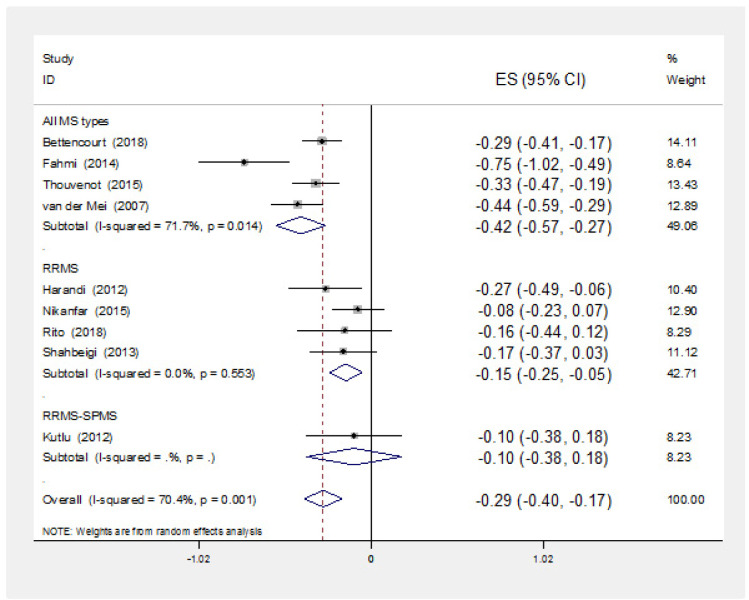
Point and pooled correlation coefficients (95% confidence intervals) between vitamin D level and disability of MS patients based on MS type in studies carried out among both sex (evidences carried out among one of sexes were limited and did not mentioned in this graph). ES: Effect Size.

Results of subgroup analysis based on MS type are illustrated in figure 3. Of four studies reported the results of all MS types, all of them reported negative and significant correlation coefficients. Combining the results of these four evidences, the pooled correlation coefficient was estimated as of −0.42 (−0.57, −0.27). Four studies reported correlation coefficients among PRMS patients all of which were negative. Just one of these correlations was statistically significant. The pooled correlation coefficient of these four evidences was estimated as of −0.15 (−0.25, −0.05). One of these evidences had been carried out among PRMS and SPMS patients reported negative non-significant correlation coefficient (Figure 3).

Egger test showed no evidence of publication bias (p = 0.217). In addition, sensitivity analysis revealed that all primary studies had similar effect on the total estimate.

**Table 1.** Characteristics of primary studies included in meta-analysis.

First author/year	Country	25 (OH) vitamin D measurement method	Type of MS	Sex	Mean age	SD age	sample size	Correla-tion	P-value	Quality score (of 9)
Bettencourt 2018 (21)	Portugal	Electrochemiluminescence immunoassay (ECLIA)	All types	F/M	41.1	11.3	244	−0.293	<0.001	7
Brola 2016 (17)	Poland	Chemiluminescent immunoassay	RRMS	F	36.4	8.2	122	−0.38	0.001	5
Fahmi 2014 (14)	Egypt	EIA Kit (Immundiagnostik)	All types	F/M	-	-	25	−0.755	<0.001	7
Faragoso 2017 (22)	Brazil	Chemiluminescent immunoassay	All types	F/M	35	-	535	-	0.39	5
Harandi 2012 (23)	Iran	High-performance liquid chromatography	RRMS	F/M	33.9	9.2	78	−0.273	0.016	7
Karampoor 2016 (24)	Iran	Chemiluminescent immunoassay	All types	F/M	41	10.2	700	-	0.34	5
Kragt 2009 (25)	Netherlands	Competitive protein binding assay	All types	F/M	45.7	10.6	101	-	>0.05	6
Kragt 2009 (25)	Netherlands	Competitive protein binding assay	All types	M	45.8	-	33	-	>0.05	6
Kragt 2009 (25)*	Netherlands	Competitive protein binding assay	All types	F	45.3	-	68	−0.25	0.044	6
Kragt 2009 (25)**	Netherlands	Competitive protein binding assay	All types	F	45.3	-	68	−0.29	0.020	6
Kutlu 2012 (15)	Turkey	ELISA kit (immunodiagnostic systems)	RRMS-SPMS	F/M	37.96	8.09	50	−0.10	-	7
Nikanfar 2015 (16)	Iran	Chemiluminescent immunoassay	RRMS	F/M	33.6	7.69	168	−0.08	0.280	7
Rito 2018 (26)	Mexico	Chemiluminescent immunoassay	RRMS	F/M	33.1	8.2	50	−0.159	0.270	8
Rito 2018 (26)	Mexico	Chemiluminescent immunoassay	RRMS	F	32.2	8.8	29	−0.521	0.001	8
Rito 2018 (26)	Mexico	Chemiluminescent immunoassay	RRMS	M	34.3	8	20	0.329	0.620	8
Shahbeigi 2013 (27)	Iran	High-performance liquid chromatography	RRMS	F/M	34.19	9.1	98	−0.168	0.049	7
Thouvenot 2015 (28)	France	Not mention	All types	F/M	45.3	13.1	181	−0.33	0.001	7
Van der Mei 2007 (29)	Australia	Radioimmuno assay	All types	F/M	43.5	9.3	136	−0.44	<0.01	7
Zamzam 2016 (30)	Egypt	Sandwich enzyme linked imunosorbent assay	All types	F/M	30.2	8.2	111	-	<0.001	5

Note: * Summer, ** winter.

## 4. Discussion

In this systematic review and mea-analysis, the association between vitamin D level and disability of MS according with EDSS was investigated. 19 evidences were extracted from of 14 eligible studies, so that in 13 studies were investigated between vitamin D level and disability among both sexes, as well as female were estimated as of −0.29 and −0.35 respectively. None of the evidence carried out among male showed significant results.

Bettencourt et al. [21] showed that correlation between vitamin D and disability was strongly negative and significant. In this study, the adjusted association was significant after controlled the effect of confounders. Brola et al. [17] reported the association between vitamin D and disability among both sexes in summer and winter. However, just the correlation coefficient among women in winter was extractable which was statistically significant. The mentioned coefficient among women in summer and also among men in both seasons could not be extracted, because just non-significant p value had been reported. Therefore, the results might be prone to bias.

Furthermore, the association between vitamin D and disability among both sexes, male and female in summer and winter had been reported by Kragt et al. [25]. They reported that this correlation among men in both seasons were non-significant, but did not report p value or correlation coefficient. However, the significant correlation coefficient was reported among women in these seasons. The risk of bias is probable due to failure to report of correlation coefficients for both sexes. It should be noted that based on the results of Brola [17] and Kragt [25] studies, sex can play a considerable role in the association between vitamin D and disability.

Harandi et al. study [23] was carried out among both sexes and showed significant correlation between vitamin D and disability but the results cannot be extractable for each sex. However, they reported a negative correlation just for women. Although, Karampoor et al. [24] did not report the correlations, they mentioned just a non-significant association (p = 0.34). Furthermore, Kutlu et al. study [15] was carried out among both sexes. It should be noted that both studies had been carried out among both sexes but the results did not report based on each sex. In Nikanfar et al. study [16], a non-significant association was reported.

The target population in Rito study [26] was both sexes and correlation coefficients for all samples as well as men were not significant. Shahbeigi et al. [27] investigated the correlation between vitamin D and disability among both sexes. Just the correlation coefficient for total was extractive and borderline significant. A significant correlation between vitamin D and disability in all study population (both sexes) had been reported in Thouvenot study [28]. However, sex specific correlations was not extractive.

Van der Mei [29] and Zamzam [30] studies were carried out among both sexes but results were not reported based on each sex. Both studies reported negative and significant correlations. It should be noted that correlation coefficient was not reported in Zamzam [30] study.

The prevalence and incidence of MS is higher for female than male (ranging from 1.1:1 to 3:1 in the majority of European studies) [7] and its growing rate, over the last decades, was faster in female than in male [7],[32], but male relapse-onset patients accumulate disability according EDSS faster than female patients while the rate of disability accumulation between male and female patients with primary progressive MS was similar [33]. As far as, DBP has additional roles more than bonding 25 (OH) vitamin D and other metabolites, such as transporting fatty acids, protecting complement C5a from degradation, macrophage activation, neutrophil chemotaxis, and functioning as an actin scavenger [34],[35], so that it can critical role on pathogenesis of MS [36]. Because DBP had been 3 main phenotypic variants that can alter 25 (OH) vitamin D levels which altered DBP levels in different race [34],[37]. Sollid et al. showed that total 25 (OH) vitamin D and DBP concentrations were lower in subjects with the Gc2/Gc2 phenotype compared to phenotypes with the Gc1S allele, and lower in males than in females [38]. Some conditions alter DBP level, estrogen and growth hormone up regulate DBP but hyperparathyroidism, obesity and insulin resistance down regulated its [34]. Furthermore, chronic conditions such as cirrhosis, nephritic syndrome decreased DBP and albumin level but free 25 (OH) vitamin D level was not alter. [39],[40]. Therefore, this situations can altered DBP and albumin level. However more than 99% of 25 (OH) vitamin D bonded with DBP and albumin (85–90% and 10–15%, respectively), but unbound hormone is biologically active which must measurement with precision method [31].

Furthermore, it seems that hormone-related physiological conditions due to sex differences in females such as puberty, pregnancy, puerperium, and menopause imply significant influence on disease prevalence and outcomes so that earlier disease onset and more frequent relapses took place in females; and faster progression and worse outcomes in males. Because of the presence of hormone receptors on immune cells witch they can inﬂuence different aspects of immune system function, potentially affecting risk, activity, and progression of MS. As far as, MS is autoimmune disease witch inﬂuenced by environmental and/or genetic factors via complex interactions occurring between sex hormones, sex chromosomes, and immune response genes [32]. Furthermore, studies shown that estrogen has been increased expression of vitamin D receptor and enhance vitamin D function, thus anti-inflammatory response in females was more potent than males. However, estrogen-mediated effects on immune response may regulate via Th1 or Th2 profile, depending on hormone concentration [41]. Luchetti et al., according of changing gene expression and inflammatory cytokines in MS lesions of male and female patients, shown that sex differences in the CNS of MS patients may affect pathogenesis, so that this changes, in males due to estrogen synthesis and its signaling; whereas in females due to progestogen synthesis and its signaling. They proposed that the contribution of this factors can lead to sex differences in the prevalence and course of MS [42].

Fahmi et al. [14] found strong correlation between vitamin D and disability. They also reported a negative correlation between vitamin D and duration of the disease. While an association was observed between duration and severity of this disease. Conversely, no significant association was reported between vitamin D and severity or duration of MS in the study carried out by Faragoso et al. [22]. Therefore, adjusting the effect of disease duration can show better association between vitamin D level and disability. Also, Nikanfar et al. study show that vitamin D level was significantly associated with duration of MS. Vitamin D has Immuno-regulatory effects and therapeutic potential in MS that increased production of the anti-inflammatory cytokine such as IL-10 [34]. Hashemi et al. shown that sufficient serum levels of vitamin D_3_ not only can increased production of IL-10 witch has anti-inflammatory effect but also decreased levels of IL-17A and IL-6 witch has pro-inflammatory effect [43]. Furthermore, the neuroprotective eﬀect of vitamin D in the cognitive decline of aging has recently been reported. Additionally roles of vitamin D reported in several aging conditions, including cognitive decline and neurodegeration [44]. Behrens et al. show that DBP levels was not different between healthy controls and patients with MS but calculated 25 (OH) vitamin D levels was lower in MS patients [45]. It is clear that further studies are needed to delineate the levels of DBP and free 25 (OH) vitamin D in patients with MS.

The present meta-analysis showed a significant negative correlation between vitamin D level and disability but not correlate with number of RRMS meanwhile vitamin D level of RRMS was lower than SPMS. Although, another review study [46] reported that lower vitamin D levels had correlated with other surrogates of MS disease activity, including lower odds of RRMS [47],[48], greater disability and disease severity in MS [27],[47],[49], conversion from clinically isolated syndromes to clinically definite MS [50].

One of the strengths of the current meta-analysis is the strong pooled effect measure of the association between vitamin D and disability of MS according with EDSS. Because we extracted data from primary studies, we reported which method was used for measurements of 25 (OH) vitamin D in each study. Free 25 (OH) vitamin D level was not directly me seared on the each study. Methods were used for determination of 25 (OH) vitamin D overestimated its level that can biased on correlation of 25 (OH) vitamin D and disability among patients with multiple sclerosis, however deficiency had correlation with disability. Therefore this bias cannot reverse conclusion of study. Language bias is one of the limitations of this study so that evidences probably published in languages other than English were missed from this meta-analysis. It should be noted that no evidence of publication bias was observed in our study. The primary studies had not reported the correlations based on age and disease duration. Another limitation was that the correlations were not reported based on each sex and MS type in all primary studies. Therefor we did not perform subgroup analysis based on age and disease. In addition, correlation coefficients and P values were not reported in all primary studies. Climate and geographical area can be the potential confounders of the correlation between vitamin D and disability. Because skin synthesis of vitamin D is associated with these situations. Furthermore, seasonal alteration in vitamin D level can be confider, however two studies design were according to summer and winter and we were considering this category when done data analysis. Several confider such as: vitamin D supplementation, sun exposure, geographical latitude and smoking effect on MS due to alteration of vitamin D level. Effect of other confider, such as: treatment, age, disease severity, genetic, disease duration, weigh, Epstein–Barr virus (EBV) infection, obesity and smoking, were not due to alteration of vitamin D level.

## 5. Conclusions

Our meta-analysis showed negative and significant relationship between vitamin D level and disability among MS patients indicating decrease in the severity of the disease by increasing the vitamin D level. Although limited evidences carried out among male, the two studies reporting these associations in this sex was not significant. It is recommended to ad regular monitoring and improvement of vitamin D levels in MS patients in the MS control and surveillance program. These studies should also consider the above limitations of the current meta-analysis and investigate the associations based on sex, age, and MS type and disease duration.
